# Recent advances in investigation of circRNA/lncRNA-miRNA-mRNA networks through RNA sequencing data analysis

**DOI:** 10.1093/bfgp/elaf005

**Published:** 2025-04-19

**Authors:** Yulan Gao, Konii Takenaka, Si-Mei Xu, Yuning Cheng, Michael Janitz

**Affiliations:** School of Biotechnology and Biomolecular Sciences, University of New South Wales, Gate 11 via Botany St, Sydney, NSW 2052, Australia; School of Biotechnology and Biomolecular Sciences, University of New South Wales, Gate 11 via Botany St, Sydney, NSW 2052, Australia; School of Biotechnology and Biomolecular Sciences, University of New South Wales, Gate 11 via Botany St, Sydney, NSW 2052, Australia; School of Biotechnology and Biomolecular Sciences, University of New South Wales, Gate 11 via Botany St, Sydney, NSW 2052, Australia; School of Biotechnology and Biomolecular Sciences, University of New South Wales, Gate 11 via Botany St, Sydney, NSW 2052, Australia

**Keywords:** circRNA/lncRNA-miRNA-mRNA, RNA sequencing, RNA networks, transcriptome, competitive endogenous RNAs

## Abstract

Non-coding RNAs (ncRNAs) are RNA molecules that are transcribed from DNA but are not translated into proteins. Studies over the past decades have revealed that ncRNAs can be classified into small RNAs, long non-coding RNAs and circular RNAs by genomic size and structure. Accumulated evidences have eludicated the critical roles of these non-coding transcripts in regulating gene expression through transcription and translation, thereby shaping cellular function and disease pathogenesis. Notably, recent studies have investigated the function of ncRNAs as competitive endogenous RNAs (ceRNAs) that sequester miRNAs and modulate mRNAs expression. The ceRNAs network emerges as a pivotal regulatory function, with significant implications in various diseases such as cancer and neurodegenerative disease. Therefore, we highlighted multiple bioinformatics tools and databases that aim to predict ceRNAs interaction. Furthermore, we discussed limitations of using current technologies and potential improvement for ceRNAs network detection. Understanding of the dynamic interplay within ceRNAs may advance the biological comprehension, as well as providing potential targets for therapeutic intervention.

## Introduction

### Importance of non-coding RNAs in gene regulation

Transcriptome analyses have revealed that up to 90% of transcripts do not encode proteins and are termed non-coding RNAs (ncRNAs) [1]. A growing number of studies across cell types, tissues and diseases have shown ncRNAs’ function as gene regulators at transcriptional and translational levels, as well as functioning in cell development and differentiation across many cell species [2–8]. With the identification of a large fraction of non-protein-coding transcripts, ncRNAs are categorized into small ncRNAs or long non-coding RNAs (lncRNAs) based on the length of sequences and genome organisation [8]. Small ncRNAs are grouped into categories based on their biological and biochemical characteristics; small interfering RNAs (siRNAs), micro RNAs (miRNAs), Piwi-interacting RNAs (piRNAs). miRNAs, which are ~25 nucleotides (nts) in length, will be a focus in this review due to its regulatory significance of translational repression and mRNA decay when interacting with other lncRNAs and the target mRNAs. lncRNAs are over 200 nt in length and act in gene regulation by interlinking networks with ncRNAs and protein-coding genes [9, 10]. Among the more than 16,000 lncRNA genes, the majority of lncRNAs were transcribed by RNA polymerase II (Pol II) [11], but Pol I and Pol III promoters have also been identified in relation to lncRNA expression. As Pol II is responsible for both lncRNAs and mRNAs transcriptions, these transcripts share similar features including a 5′ cap and poly(A) tail, which are crucial to the stability of a transcript and nuclear export. One of the most important lncRNAs biogenesis discussed in this review is the long intergenic RNAs (lincRNAs) which are lncRNAs expressed from the intergenic region of the protein-coding gene [11, 12]. Another type of ncRNA known as circular RNAs (circRNAs) (250 nt–4 kb) was first visualized in a covalently closed circular conformation in the 1970s [13, 14]. The circular loops are commonly facilitated by lariat-driven circularisation, known as the exon-skipping mechanism [3]. Once the 5′-donor site of an intron attacks the 3′-acceptor site of the targeted intron, a lariat is formed as a by-product from the splicing event. This lariat contains the skipped exons and introns, and the introns can be removed or retained for exonic circRNAs or exon-intron circRNAs generation [2, 15]. Investigation of different types of ncRNAs has offered insights into the interaction between ncRNAs and coding RNAs, and the gene regulatory system behind this interaction. Furthermore, lncRNAs and circRNAs are termed as competitive endogenous RNAs (ceRNAs) due to the discovery that these RNA molecules offer gene regulation by competiting for miRNAs [16–18]. The interconnection between the transcripts promotes the growing number of studies and subsequent bioinformatics tools to be developed. Such investigation of the contribution of ceRNAs to the understanding of evolutionary biology and the regulatory roles in disease pathogenesis [19].

### Overview of ceRNAs and the analytical workflows for identification of ceRNA networks using RNA sequencing data

**Figure 1:**
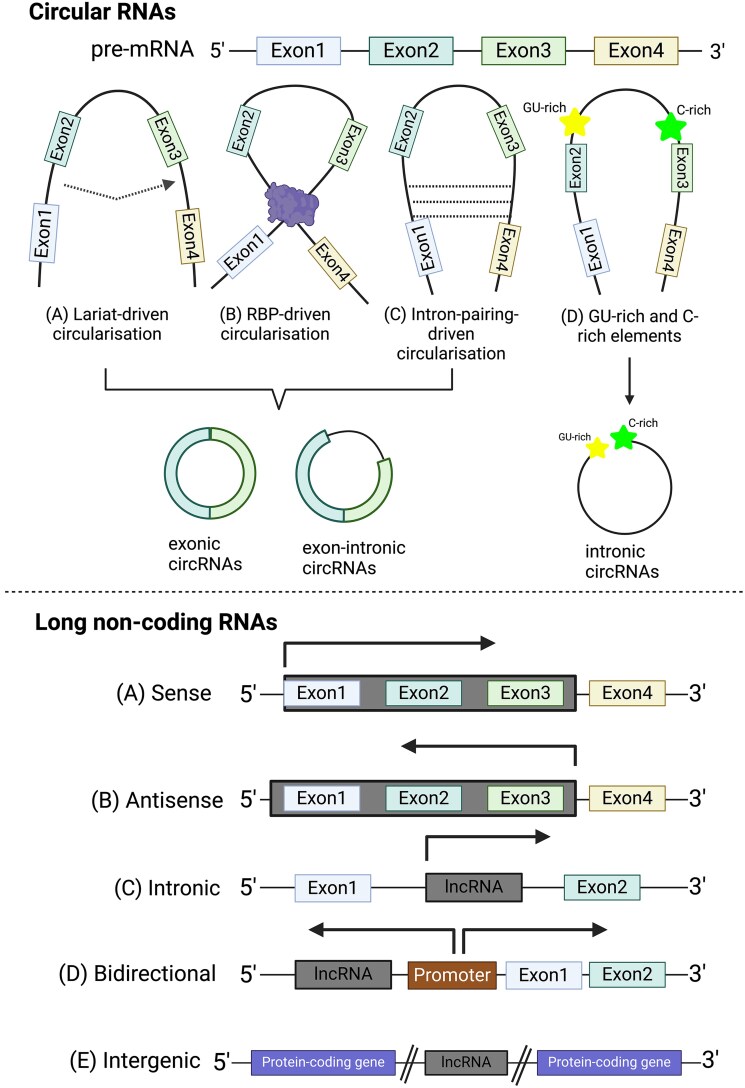
Biogenesis of circRNAs (top) and lncRNAs (bottom). CircRNAs biogenesis (top): From pre-mRNA, (A) the initial lariat can be formed through the interaction of the 5′-donor site and 3′-acceptor site, (B) the dimerisation of the RNA-binding proteins (RBPs) locating on the downstream and upstream motifs, (C) the interaction of the flanking sequences on introns, (D) or interaction of the GU-rich and C-rich elements. The involvement of exons and introns further classifies mature circRNAs. LncRNA biogenesis (bottom): Sense (A) and antisense (B) lncRNAs are transcribed from the sense and antisense strand of the gene, respectively. (C) Intronic lncRNAs are entirely transcribed from the intron region. (D) Bidirectional lncRNAs are transcribed from the same promoter of another transcript but in an opposite direction. (E) Intergenic lncRNAs are located in-between two protein coding genes.

In this review, circRNAs and lncRNAs are the primary focus and how they are identified as ceRNAs to regulate miRNAs and downstream transcripts progressively will be explored. CircRNA, a unique class of ncRNAs, is formed from a covalently closed loop by the ligation of downstream and upstream sites of exons or introns [20]. Mature circRNAs are formed with a process described as back-splicing, and the distinct factors involved during the back-splicing event modulate the development of exonic circRNAs, exonic–intronic circRNAs or intronic circRNAs (Fig. 1). Noticeably, current circRNA detection relies on back-splice junctions (BSJ) sites which are the edges of the ligated exons or introns [21]. The mature circRNAs have been identified to be highly stable and evolutionarily conserved due to their covalently closed circular structure that consists of the lack of 5′ cap and 3′ poly(A) tail, thereby allowing them to be resistant to exonuclease degradation. RNA sequencing (RNA-seq) is an essential and widely applied next-generation sequencing technology, widely used in transcriptomic studies [22]. The application of RNA-seq data and bioinformatics tools has enhanced the understanding on transcriptomic profiling of ncRNAs and ceRNAs network in recent years. As non-polyadenylated ncRNAs, circRNAs can be retained through the ribosomal RNA (rRNA) depletion during the library preparation for RNA-seq [3]. LncRNAs are categorized based on the direction of transcription and whether the transcribed lncRNA overlaps with protein-coding genes (Fig. 1) [8]. Identification of lncRNAs through bioinformatics tools is based on the criteria such as protein-coding ability, transcripts structure and open reading frames (ORF) [23]. On the other hand, lncRNAs of which are non-polyadenylated can be distinguished from polyadenalyted lncRNAs and mRNAs through poly(A)-seq that is performed by RNA-seq based on their presence of poly(A) tails at the 3′ end of the strand [8, 24]. Furthermore, long-read sequencing technology enhances the detection and characterisation of RNAs molecules by capturing full-length sequences without fragmentation of the RNA transcripts, thereby improving the accuracy of the transcriptome profiling [25].

**Figure 2:**
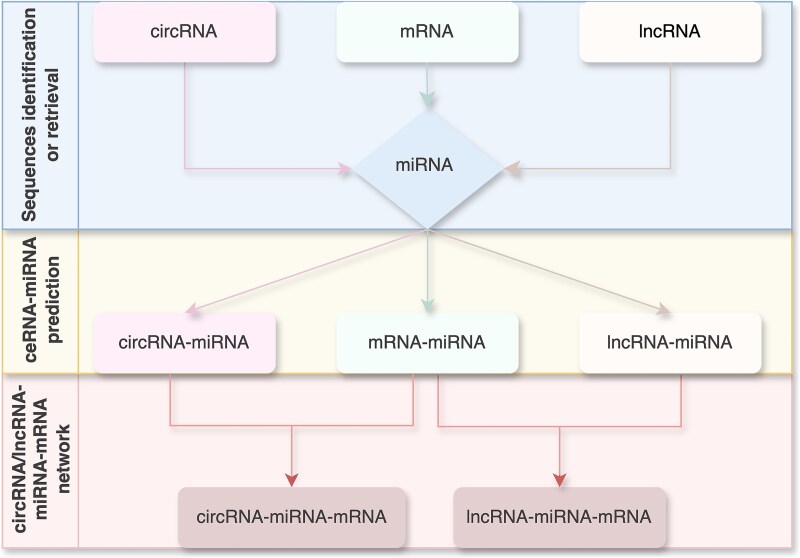
circRNA/lncRNA-miRNA-mRNA network prediction flowchart. The first step involves collecting sequence data for the four RNA molecules. Then the interaction between each ceRNA and miRNA are predicted based on the computational analysis using sequence data. The final step integrates the predicted interactions into comprehensive ceRNAs networks.

Prediction of a ceRNAs network by using RNA-seq data and computational programs necessitates stepwise identification. A great number of bioinformatics tools and databases have been developed to specifically annotate, characterize, and/or store reposit the genomic information of circRNAs, lncRNAs, mRNAs and miRNAs [26–29]. Of note, small ncRNAs species necessitate specific small RNA-seq (sRNA-seq) method due to their short sequence length [22]. Intermediate networks are constructed for individual ceRNA according to miRNAs through scanning the potential miRNA binding sites (Fig. 2). The commonly detected miRNAs mediate the indirect regulation between circRNAs and mRNAs, or between lncRNAs and mRNAs [30–32].

## Biological significance of ceRNA-miRNA networks

### miRNAs influence translational repression and mRNA decay

**Figure 3:**
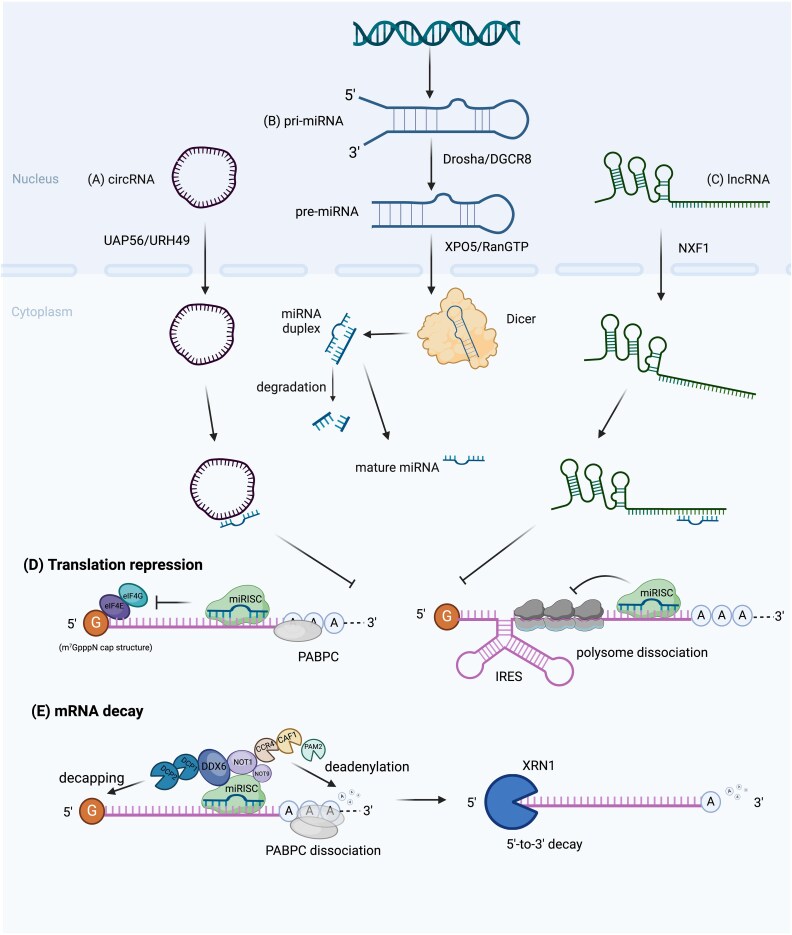
Mechanism of ceRNAs inhibit miRNA-mediated translation repression and mRNA decay. (A) Mature circRNA that is formed inside the nucleus is transported to the cytoplasm, mediated by UAP56 or URH49 proteins. UAP56 exports larger circRNAs (>1200 nt), and URH49 exports short circRNAs (<400 nt). CircRNAs located in the cytoplasm complementarily binds to mature miRNA with MREs. (B) Pri-miRNA is transcribed by the RNA polymerase II in the nucleus, which is then cleaved to become pre-miRNA by Drosha and DGCR8 before being exported to the cytoplasm by XPO5 and RanGTP. The shorter strand of the miRNA duplex is processed by a Dicer and degraded, while the other strand develops into a mature miRNA. (C) Transportation of the lncRNA is similar to mRNAs, but is highly dependent on the NXF1 complex. The MREs on lncRNA mediate complementary binding to miRNA. The interaction between circRNA-/lncRNA- inhibit miRNA from binding to the target mRNAs for translation repression and mRNA decay. (D) The target mRNA sequence is recognized by miRISC by base-pairing, which interferes the interaction between initiation factors and cytoplasmic poly(A) binding protein (PABPC), as well as lead to polysomes disassemble. (E) The deadenalyse complex (CAF1-CCR4-NOT) promotes mRNA deadenylation, eventually resulting in decapping mediated by decapping proteins DCP1/2 complex, PABPC dissociation, and exonucleolytic decay by XRN1.

Based on the Watson-Crick concept of complementarily pairing bases, miRNAs recognize their mRNA targets through the complementary binding sites located at 3′-untranslated region (UTR) of the mRNAs [33]. The discovery of the miRNA-targets correlation have expedited experimental and computational analysis, thereby promoting the contribution of miRNAs in translation repression and mRNAs targets degradation [34, 35]. Although mRNA translation requires multiple factors and sufficient ribosomal subunits for proper initiation, elongation and termination, previous studies demonstrated that the presence of miRNAs lead to the shift of mRNAs towards where less ribosomes were involved or undergo cap-independent translation initiation at the internal ribosome entry sites (IRES) [36]. Due to the variation of cap structures on mRNA sequences, there are reports of miRNAs which inhibit mRNAs with the m^7^GpppG (m^7^G) cap structure but not with ApppG cap structure at the initiation of translation (Fig. 3) [37]. A finding revealed that the initiation factor, eIF4G, was suppressed by miRNA-induced silencing complex (miRISC) during the recognition of m^7^G cap process [38]. MiRNAs were previously reported to interact with Ago proteins and RISC to mediate translational repression [39]. Moreover, early studies have shown that miRNAs’ silencing machinery could direct deadenylation of mRNA poly(A), leading to poly(A)^−^ mRNAs which are less prone for translation [39, 40]. At post-initiation stage, mRNAs associated with IRES-dependent translation can also be repressed when miRNAs dissociate polysomes (also known as polyribosomes) from the polypeptide chain, which eventually lead to termination of translation process [34, 41]. The participation of miRNAs in mRNA target degradation proceeds from the CAF1-CCR4-NOT deadenylase complex recruited by miRNAs. Subsequentially, the decapping enzyme DCP2 and cytoplasmic 5′-3′ exonuclease XRN1 are loaded for ultimate mRNA decay [34].

The ceRNAs hypothesis proposed the regulatory function of coding and non-coding RNAs are mediated by the shared miRNA binding sites [19]. The interaction between ceRNAs and miRNAs occurs mostly in cytoplasm [42, 43]. The ncRNAs are able to act as ceRNAs to sequester the miRNA-mediated activity and consequently re-activate the expression of the transcripts originally targeted by miRNAs (Fig. 3) [7, 44, 45]. It has been noted that miRNAs interact with their targets through binding to miRNA response elements (MREs) [33]. To highlight the functional relevance of conservation, many MREs within ceRNAs are highly conserved across evolutionary lineage [3, 19, 46]. Hence, circRNAs and lncRNAs play roles of miRNAs sponges with the presence of MREs, thereby inhibiting the miRNAs from binding to the 3’-UTR of the linear RNA transcripts. While the miRNAs are captured by circRNAs or lncRNAs, activities including the translation repression and mRNA decay that are promoted by miRNAs are suppressed.

### Function of ceRNAs in post-transcriptional, translational repression and mRNA decay

Several examples that are associated with circRNAs or lncRNAs are mentioned in this review based on our current understanding of miRNA-mediated translational repression and mRNA decay. Across several studies, antisense to the cerebellar degeneration-related protein 1 transcript (CDR1as) act as ceRNA to sponge and antagonize many miRNAs to further regulate miRNAs’ repression ability [3, 45, 47, 48]. Knockdown of CDR1as correlates with the decrease of EGFT and IFT1R mRNA expression, since miR-7 becomes available to bind to the targets and interfere their expression [49]. Additionally, the silenced CDR1as allows miR-7-5p to significantly reduce the mRNA and protein expression of E2F transcription factor 3 (E2F3), which eventually restrains the cell growth and glucose metabolism [50]. It was previously discovered that the interference of circHIPK3 can mediate miR-30a-3p to limit myocyte enhancer factor 2 C (MEF2C) expression in terms of myoblast [51]. Analysis of inhibited circ101491 demonstrated a co-inhibition effect of oncogene endothelin1 (EDN1) as miR-125b-5p effectively represses the target expression [52]. Down-regulation of circ_0084043 and up-regulation of miR-429 leads to a decrease in tribbles homolog 2 (TRIB2) mRNA and protein expression [53].

By targeting MREs, lincRNAs are regulated by miRNAs for further manipulation on mRNAs expression [7, 19]. It was shown that knockdown of LINC00173 facilitates decreased enrichment of Ago2 loading, which subsequently prevented the Etk from miR-218-mediated mRNA decay in both the nucleus and the cytoplasm [42]. In another study, LINC00173 was shown to be down-regulated pattern in lung adenocarcinoma (LUAD), which significantly contribute to down-regulate ZFP36L2 expression while the activity of miR-1275 towards the target expression was not hindered by LINC00173 [54]. As ZFP36L2 can promote mRNA degradation and protein translation inhibition regarding its structure of the CCCH zinc-finger and binding to the AU-rich elements in the 3’-UTR of target mRNA, BCL2 transcripts become vulnerable as a target in LUAD [55]. Intriguingly, down-regulation of ZFP36L2 in the research allows for the BCL2 mRNA to enhance stability, which suppresses cell apoptosis consequentially.

### Roles of ceRNAs in disease, development, and differentiation

In the past decades, a growing number of research has shown that the ceRNAs networks are involved in biological processes across many diseases (Table 1). The most significantly novel role of ceRNAs is acting as miRNAs sponges which subsequentially regulates target gene expression in diseases [56–58]. In breast cancer, circPGR was found to regulate the expression cell cycle genes by sponging miR-301a-5p, which promoted the estrogen-receptor-positive breast cancer cell growth [56]. Another study demonstrated that an up-regulation of circPVT1s are involved in endometrial cell proliferation and invasion through negatively regulating miR-145 and positively regulating Talin1 protein in adenomyosis [59]. The ceRNA network constructed with the miR-145-5p, which originated from the 5′ end, was identified as a marker for differentiating old adipose-derived stem cells (O-ASCs) from ASCs phenotypes [60]. Reduction of the lncRNA RAET1E-AS indicated that less miRNA sponges were free to interact with miR-145-5p, therefore, overexpression of miRNAs could promote cell proliferation and rejuvenate cellular senescence. As an immune regulator, a newly identified intronic circRNA, circAIVR, was able to stimulate IFN-β from the immune system to function as an antiviral factor *via* sponging miR-330-3p and enhance the expression of CREBBP protein in the influenza virus-infected cells [61]. While ACTB protein serves in vascular remodelling, down-regulation of ACTB in hypertrophic cardiomyopathy significantly affects the progression of this cardiovascular disease [62]. Down-regulation of lncRNA XIST and CAMD1 in atrial fibrillation disease were indicated to promote the dysregulation of proliferation of fibrosis-related proteins and PI3K/AKT pathway correlated with fibrosis, respectively [63]. Peripheral blood tests from type 2 diabetes mellitus patients predicted an association between vascular injury and the competitive lncRNA KCNQ1OT1/circ_0020316-miR-92a-2-5p-MAPK3 axis [64]. Alzheimer’s disease (AD) is a neurodegenerative disease, in which reports of the lncRNA SNHG10/OIP5-AS1-miR-3158-3p-RPL35A network contributed to AD pathogenesis *via* affecting the amyloid precursor protein [57]. From the hippocampus brain region of Down Syndrome (DS) patients, up-regulation of lncRNAs RMST and GART were observed, and miR-548b-5p/miR-548ad-5p/miR-144-3p were found to be pivotal in regulating the target gene GART expression [65]. Therefore, the researchers stated that GART might serve as a potential biomarker for prenatal blood diagnosis for neural defects in DS [65].

**Table 1:** lncRNA/circRNA-miRNA-mRNA networks in development, differentiation, and disease.

	Disease	lncRNA/circRNA	Change	miRNA	Expression	mRNA	Change	Functions	Year	Ref.
1	Breast cancer	circPGR	Up	miR-301a-5p	Down	CDK1/CDK6/CHEK2	Up	Regulation of cell cycle progression, cell growth	2021	[56]
2	Adenomyosis	circPVT1	Up	miR-145	Down	Talin1	Up	Eutopic endometrial cell proliferation	2021	[59]
3	Mesenchymal stem cells	lncRNA RAET1E-AS1	Down	miR-145-5p	Up	WNT11/BMPER	Down	ASC proliferation	2021	[60]
4	Influenza virus infection	circAIVR	Up	miR-330-3p	Down	CREBBP	Up	IFN-β production	2021	[61]
5	Hypertrophic cardiomyopathy	lncRNA ADAMTS9-AS1/ circFN1	Down	miR-206	Up	ACTB	Down	HCM progression	2022	[62]
6	Kaposi’s sarcoma-associated herpesvirus	hsa_circ_0070049/lncRNA AL031123.1	Up	hsa-miR-378i	Down	SPEG/FOXQ1	Up	Inhibition of KSHV lytic replication	2022	[66]
7	Atrial fibrillation	XIST/circRNA_2773	Down	miR-486-5p	Up	CADM1	Down	AF pathogenesis	2023	[63]
8	Type 2 diabetes mellitus	lncRNA KCNQ1OT1/circ_0020316	Up	miR-92a-2-5p	Down	MAPK3	Up	Vascular injury	2023	[64]
9	Calcific aortic valve disease	hsa-circ-0073813/hsa_circ_0027587	Up	hsa-miR-525-59	Down	SPP1/HMOX1/CD28	Up	CAVD pathogenesis	2023	[67]
10	Alzheimer’s disease	lncRNA SNHG10/OIP5-AS1	Down	hsa-miR-3158-3p	Up	RPL35A	Down	AD pathogenesis	2023	[57]
11	Bladder cancer	lncRNA LINP1/hsa_circ_0075881	Up	hsa-324-3p	Down	ST6GAL1	Up	Development of bladder cancer cell resistance to gemcitabine	2023	[68]
12	Down syndrome	lncRNA RMST	Up	miR-548b-5p/miR-548ad-5p/miR-144-3p	Down	GART	Up	Regulatory role in occipital cortex and cerebellum	2024	[65]
13	Obesity type 2 diabetes	hsa_circ_0060614	Up	hsa-mir-4668-3p	Down	MT2A	Up	Diabetes-related metabolic disorders	2025	[69]

Gene name retrieved from https://www.ncbi.nlm.nih.gov/gene/: PGR, progesterone receptor; CDK1/2 cyclin dependent kinase 1/2; CHEK2 checkpoint kinase 2; PVT1 PVT1 oncogene; RAET1E RAE1E antisense RNA 1; WNT11 Wnt family member 11; BMPER BMP binding endothelial regulator; CREBBP CREB binding protein; ADAMTS9-AS1 ADAM metallopeptidase with thrombospondin type 1 motif 9 antisense 1; FN1 fibronectin 1; ACTB actin beta; SPEG striated muscle enriched protein kinase; FOXQ1 forkhead box Q1; XIST X-inactive specific transcript; CADM1 cell adhesion molecule 1; KCNQ1OT1 potassium voltage-gated channel subfamily Q member 1 opposite strand/antisense transcript 1; MAPK3 mitogen-activated protein kinase 3; SPP1 secreted phosphoprotein 1; HMOX1 heme oxygenase 1; CD28 CD28 molecule; SNHG10 small nucleolar RNA host gene 10; OIP5-AS1 opa interacting protein 5 antisense 1; RPL35A ribosomal protein L35a; LINP1 lncRNA in non-homologous end joining pathway 1; ST6GAL1 ST6 beta-galactoside alpha-2,6-sialyltransferase 1; RMST rhabdomyosarcoma 2 associated transcript; GART glycinamide ribonucleotide transformylase; MT2A metallothionein 2A.

With regards to the circRNA-miRNA-mRNA network in cell development in diseases, a number of studies demonstrated the regulatory role of CDR1as-miR-7 axis across different diseases. Tang et. al [49] investigated that CDR1as blocks miR-7 which results in the up-regulation of EGFT and IFT1R which lead to progression and proliferation of colorectal cancer cells. In esophageal squamous cell carcinoma, homeobox B13 (HOXB13) is significant in tumorigenesis, overexpression of CDR1as abrogates miR-7 from suppressing HOXB13, promoting malignant progression in patients [70]. Research on adipocyte development revealed the role of circRNF111 in adipogenesis through sponging miR-27b-3p and re-activating PPARγ expression for pre-adipocyte differentiation [71]. When miR-125b-5p is interfered by the ceRNA sponge, overexpression of circ101491 and EDN1 promotes cell proliferation, invasion and metastasis of glioma cells *via* exosomes [52]. Positive correlation between circ_0084043 and TRIB2 is expressed through interfering miR-429, thereby enabling the restriction of the development of melanoma cells, cell invasion, metastasis, as well as activating downstream pathway such as Wnt/β-catenin signaling [53]. A recent computational analysis constructed a ceRNA network in obesity type 2 diabetes, revealing that the increase of hsa_circ_0060613 significantly up-regulates MT2A expression by sponging hsa-mir-466803P [69]. It has been noted that the expression of MT2A from the diabetes is clinically related to inflammatory factor IL-6 from chronic immune response and mineral homeostasis including zinc deficiency [72].

LncRNA-miRNA-mRNA cross-talk serves as a critical modulator in development and cell differentiation, which offers insights into the intricate mechanisms in a range of disease. A previous study identified that LINC00173 suppressed miR-218 in small cell lung cancer (SCLC) and upregulated N-myc downstream-regulated gene 1 (NDRG1) and GSK3β-interacting protein (GSKIP) [42]. The two target genes could facilitate β-catenin to translocate from the cytoplasm to the nucleus, ultimately driving the chemoresistance, progression and cell proliferation of SCLC. Downregulation of LINC00173 in lung adenocarcinoma was found to suppress protein interacting with cyclin A1 (PROCA1) and ZFP36 ring finger protein like 2 (ZFP36L2) as competing for miR-1275, hence, inducing apoptotic signal reduction and chemoresistance that negatively impact on cellular homeostasis in disease [54]. Wang et. al [73] revealed that lincRoR and several core transcription factors such as octamer-binding transcription factor 4 (Oct4), SRY-Box transcription factor 2 (Sox2), and nanog homeobox (Nanog) are positively correlated through the miRNA-involved mechanism in self-renewing embryonic stem cells (ESCs). The role of endogeneous lincRoR was described to be involved in genetic regulation n and physiological functions in early development of ESCs self-renewal. Zhang et.al [74] predicted that the ceRNA networks potentially are related to oxidative stress, mitochondrial function, and immune cell interaction in keloid formation. This recent study integrated multi-omics approach with machine learning method to predict that the lncRNA AC005062.1-miR-134-5p/FKBP5 and the lncRNA BASP1-AS1/miR-503-5P/ADH1B, potentially contributing to the regulation of keloid immune microenvironment.

The investigation of ceRNA networks has presented a sophisticated regulatory framework that is integral to various disease pathogenesis, development, and cell differentiation. The number of examples discussed above highlight the critical role of ceRNAs in the context of sequestering miRNAs and influencing mRNAs expression. Hence, the exploration of these ncRNAs not only enhance the understanding of fundamental biological concepts but also creates broader avenues for future therapeutic strategy [75].

## Tools and databases for ceRNAs axes prediction

The prediction and construction of circRNA/lncRNA-miRNA-mRNA network usually comprise of multiple tools and databases (Table 2). Genomic sequences of circRNAs, lncRNAs, miRNAs, and mRNAs are used to search for the miRNA target binding sites (also known as MREs) in order to predict the ceRNAs axes accordingly. This section introduces the tools and databases used to predict circRNA/lncRNA-miRNA-mRNA networks.

**Table 2:** List of tools and databases for circRNA/lncRNA-miRNA-mRNA network prediction.

Names	Functions	Tools or Databases^1^	Platform	Integrative tools^2^	Resources	Year	Refs
CIRCexplorer2	circRNA detection	Tool	Python	Yes	https://circexplorer2.readthedocs.io/en/latest/	2016	[15]
CircPro	circRNA detection	Tool	Perl	Yes	https://bis.zju.edu.cn/CircPro/	2017	[81]
circRNA_finder	circRNA detection	Tool	Perl	No	https://github.com/orzechoj/circRNA_finder	2014	[119]
circtools	circRNA detection	Tool	Python	Yes	https://docs.circ.tools/en/latest/#	2018	[79]
CIRI2	circRNA detection	Tool	Perl	Yes	https://ciri-cookbook.readthedocs.io/en/latest/CIRI2.html	2017	[26]
DCC	circRNA detection	Tool	Python	No	https://github.com/dieterich-lab/DCC	2015	[80]
find_circ	circRNA detection	Tool	Python	No	https://github.com/rajewsky-lab/find_circ2	2013	[3]
KNIFE	circRNA detection	Tool	Perl, Python, R	-	https://github.com/lindaszabo/KNIFE		
CIRCpedia	-	Database	Web	-	http://yang-laboratory.com/circpedia/	2018	[120]
circBase	-	Database	Web	-	http://www.circbase.org/	2014	[121]
Circ2GO	circRNA-miRNA prediction	Tool Database	Web, R	Yes	https://github.com/airbox11/circ2GO	2020	[83]
circAtlas 3.0	circRNA-miRNA prediction	Tool Database	Web	Yes	https://ngdc.cncb.ac.cn/circatlas/	2023	[85]
CircInteractome	circRNA-miRNA prediction	Tool Database	Web	Yes	https://circinteractome.nia.nih.gov/	2016	[86]
CircNet 2.0	circRNA-miRNA-mRNA prediction	Tool Database	Web	Yes	https://awi.cuhk.edu.cn/~CircNet/php/index.php	2021	[84]
circRNAprofiler	circRNA-miRNA prediction	Tool	R	Yes	https://github.com/Aufiero/circRNAprofiler	2020	[122]
CPAT	lncRNA detection	Tool	C Python	-	https://cpat.readthedocs.io/en/latest/	2013	[27]
CPC2	lncRNA detection	Tool	-	-	https://cpc2.gao-lab.org/	2017	[123]
FEELnc	lncRNA detection	Tool	Perl R	-	https://github.com/tderrien/FEELnc	2017	[93]
LncADeep	lncRNA detection	Tool	Python	-	https://github.com/cyang235/LncADeep/	2018	[91]
LncDC	lncRNA detection	Tool	Python	No	https://github.com/lim74/LncDC	2022	[88]
lncFinder	lncRNA detection	Tool	R	-	https://cran.r-project.org/web/packages/LncFinder/index.html	2019	[90]
LncRNA_Mdeep	lncRNA detection	Tool	Python	-	https://github.com/NWPU-903PR/lncRNA_Mdeep	2020	[89]
LncRNAnet	lncRNA detection	Tool	-	-	https://data.snu.ac.kr/pub/lncRNAnet/	2018	[92]
PLEK	lncRNA detection	Tool	Python	-	https://sourceforge.net/projects/plek/files/	2014	[94]
EPLMI	lncRNA-miRNA prediction (network based)	Tool	Matlab	No	https://github.com/TYLH/EPLMI	2018	[97]
GCNCRF	lncRNA-miRNA prediction (network based)	Tool	Python	No	https://github.com/zhaoqi106/GCNCRF	2022	[124]
JSCNCP-LMA	lncRNA-miRNA prediction	Tool	Python	No		2022	[98]
LMI-DForest	lncRNA-miRNA prediction	Tool	Python	Yes		2020	[96]
LNCipedia	-	Database	Web	-	https://lncipedia.org/	2013	[125]
miRBase	-	Database	-	-	https://www.mirbase.org/	2019	[107]
DIANA-LncBase	miRNA-lncRNA prediction	Tool Database	Web	Yes	https://diana.e-ce.uth.gr/lncbasev3	2015	[46]
DIANA-microT-CDS	miRNA target sites prediction	Tool Database	Web	Yes	https://dianalab.e-ce.uth.gr/html/dianauniverse/index.php?r=microT_CDS	2012	[109]
DIANA-Tarbase	miRNA-mRNA prediction	Tool Database	Web	Yes	https://dianalab.e-ce.uth.gr/tarbasev9	2017	[110]
doRiRNA / PicTar	miRNA target sites prediction	Tool Database	Web, Java, Perl	Yes	https://github.com/dieterich-lab/dorina https://pictar.mdc-berlin.de/	2012	[113]
miRanda	miRNA target sites prediction	Tool	C	No	https://bioweb.pasteur.fr/packages/pack@miRanda@3.3a	2003	[99]
miRDB	miRNA target sites prediction	Tool Database	Web, R	Yes	https://mirdb.org/	2019	[105, 106]
miRTarBase	miRNA target sites prediction	Tool Database	Web	Yes	https://mirtarbase.cuhk.edu.cn/~miRTarBase/miRTarBase_2022/php/index.php	2022	[108]
MirTarget	miRNA target sites prediction	Tool	R	Yes	https://github.com/kassambara/miRTarget	2019	[105]
miRWalk	miRNA-mRNA prediction	Tool Database	Web, MySQL	Yes	http://mirwalk.umm.uni-heidelberg.de/	2014 2015	[111, 112]
PITA	miRNA target sites prediction	Tool	Perl	No	https://genie.weizmann.ac.il/pubs/mir07	2007	[100]
RNA22	miRNA target sites prediction	Tool	C	No	https://cm.jefferson.edu/rna22/Interactive/	2006	[103]
RNAhybrid	miRNA target sites prediction	Tool	Web, Perl, C/C++, Java	No	https://bibiserv.cebitec.uni-bielefeld.de/rnahybrid	2006	[104]
TargetScan	miRNA target sites prediction	Tool	Web, Perl	No	https://www.targetscan.org/vert_80/	2015	[29]
CircMiMI	circRNA-miRNA-mRNA network prediction	Tool	Python	Yes	https://circmimi.genomics.sinica.edu.tw/	2022	[115]
CRAFT	circRNA-miRNA-mRNA network prediction	Tool	R	Yes	https://github.com/annadalmolin/CRAFT	2022	[116]
starBase	circRNA/lncRNA-miRNA-mRNA prediction	Tool Database	Web, MySQL	Yes	https://rnasysu.com/encori/	2013	[114]

Tools and Databases^1^: Tool performs computational analysis; Database stores existing data.

Integrative tool^2^: Integration of other tools.

### Tools and databases for circRNA identification and circRNA-miRNA prediction

The detection of circRNAs using RNA-seq datasets is dependent on identifying the BSJ-spanning reads. Current popular tools for circRNAs identification and annotation (Table 2) initiate through mapping of reads to the reference genome using STAR [76], BWA-MEM [77] or TopHat-Fusion [78] alignment approaches. CIRI2 adapts multiple seed matching with relative maximum likelihood estimation [26] while CIRCexplorer2 integrates chimeric fusion junction reads to reference gene annotations [15]. Circtools is built based on the DCC algorithm to further extend comprehensive circRNAs analysis and deliver more circRNAs characterisation [79, 80]. With integration of CIRI2, CircPro develops a three-module workflow including detecting junction reads from Ribo-Seq data [81]. While find_circ and circRNA_finder both work on *de novo* transcripts without previous gene annotations and/or exon-intron structures, evaluation for these two tools were scrutinized with comparatively low sensitivity by predicting lowest number of circRNA species [82]. Although databases are typically known for data storage and management, circ2GO, CircNet 2.0, circAtlas 3.0, CircInteractome databases integrate circRNAs and miRNAs detection tools to predict the miRNA binding sites on circRNAs [83–86]. Of note, although CircInteractome contains experimentally validated datasets, prediction of circRNA-miRNA network in CircInteractome is currently limited to *Homo sapiens* database. Furthermore, once the completed exonic–intronic circRNAs sequence are identified by any of the circRNAs identification tools, circRNA-miRNAs interaction can also be predicted by the tools and databases that are developed specifically for miRNA-targets identification, which are listed in Section 3.3.

### Tools and databases for lncRNA identification and lncRNA-miRNA prediction

Cumulative development of advanced technology enforce the integration of artificial intelligence with machine learning models into lncRNA sequences identification tools (Table 2) [87]. The most popular feature that distinguishes lncRNAs and mRNAs is the ORF. The ORF features in lncRNA transcripts represent the protein translation regulated by the bounded start codon and stop codon [28]. However, inaccurate prediction could occur due to the similarity between the ORF from lncRNAs and coding sequences (CDS) from mRNAs. Hence, ORF size and ORF coverage are considered in LncDC [88], lncRNA_MDeep [89], lncFinder [90], and CPAT [27], the intrinsic feature of entropy density profile of ORF or ORF indicator are additional ORF feature shown in LncADeep [91] and lncRNAnet [92], respectively (Table 2). Lack of comprehensive feature representation was concerning to accuracy as FEELnc only measures ORF coverage [93] and none of the ORF features are examined in PLEK [94]. PLEK assessed the algorithm with 10-fold cross-validation through training extensive large-scale transcriptomic data including human, mouse, zebrafish and frog (xenopus tropicalls). Beyond the ORF feature, newly developed tools including LncDC, LncADeep, and LncFInder all consider the property of secondary structure of transcripts for further classification [95]. All listed lncRNA identification tools apart from lncRNAnet consider the composition of genomic sequences in terms of k-mers, particularly hexamers (k-mer with length of 6). Fickett score is a method to calculate the composition and nucleotide positions, which is deliberately implemented in LncDC, LncADeep, lncRNA_Mdeep, CPAT, and CPC2. Recent advanced lncRNA-miRNA prediction tools (Table 2) are constructed using diverse machine learning models for prediction and validation. LMI-DForest scans and extracts the feature of lncRNA using DeepForest algorithm, and then uses the trained DeepForest and autoencoder model to predict the interaction probability [96]. EPLMI tool employs bipartite graph which is a graph-based two-way diffusion model to detect lncRNAs [97]. This tool initiates the prediction using Pearson correlation on the functional similarity and sequence similarity of the lncRNA-miRNA interaction. GCNCRP trains the model using graph convolutional network and an additional conditional random field to interpretate the constructed adjacency matrix of the lncRNA-miRNA. JSCSNCP-LMA identifies the potential lncRNA-miRNA pairs using spectral clustering model, and predicts the interaction using sparse matrix factorisation [98]. Among the four tools, LMI-DForest represents the greatest AUC (Area Under the Curve) with 0.9940 for five-fold cross-validation in the machine learning model, which directly reflects the optimal true positive rate, sensitivity and the significant specificity on both training and validation datasets [96].

### Tools and databases for miRNA identification and targets prediction

Commonly used miRNA targets and circRNA_miRNA interaction prediction tools include miRanda [99], TargetScan [29], PITA [100], of which all three miRNA-targets prediction tools employ complementary sequence pairing of miRNA sequences and 3’-UTR sequences of target genes as primary sequence matching (Table 2). miRanda integrates seed matching with thermodynamic stability that calculates the free energy of G-U wobbles from miRNA-target interaction as well as an evaluation of conservation of known miRNA-target that filters the prediction scores [99]. Beyond seed matching, context-plus model has been trained to consider and score the feature of target site type, 3′-supplementary pairing, local AU content and distance to the closest 3’-UTR ends *etc* [101]. In miRNA-target match assessments, miRanda adopts predictive power and sensitivity and avoids eluding the high false-positive rate [99]. TargetScan upgraded context++ model for high-reliability for conserved miRNA binding sites to improve the predictive power by iteratively scoring and stepwise predicting the potentials [29]. The statistical significance of the 14 features that have been implemented in TargetScan is based on 3’-UTR length, ORF length, structural accessibility, number of offset 6mer in 3’-UTR sites, number of 8mer sites in the ORF, nucleotide identify at position 8 of the target and miRNA, site conservation, updated miRNA target-site abundance, and updated seed-pairing stability [29, 102]. Comparing to miRanda, PITA defines the thermodynamic score of miRNA-target interaction by calculating the difference between the free energy of the duplex formation and the free energy of the unpairing energy [100]. Of note, both TargetScan and PITA refer to local site accessibility through focusing on either contextual factors or calculating the energy cost of accessibility in 3’-UTR, which predicts whether miRNA could interact with the target gene even under secondary structure of the mRNA. The RNA22 tool predicts the miRNA-target interaction based on their statistically significant pattern recognition [103]. The RNAhybrid online tool offers customisation of relevant parameters for seed matching requirement and hybridisation of miRNA-target sequences, as well as integrating the features of disallowance of G-U base pairs in the seed region [104]. Importantly, RNAhybrid and miRanda have extended their predictions with multiple species including not only human/mice datasets but also drosophilia, zebrafish, xenopous, worm and chicken. MirTarget is highlighted with implementation of machine learning model using support vector machine (SVM) framework for miRNA-target features prediction [105]. Through performing the trained recursive feature elimination (RFE) analysis based on SVM, the least important features can removed from each process of iteration, followed by the ranking of several seed conservation features. Furthermore, miRDB, an online customisable miRNA-target prediction database, is incorporated with the MirTarget algorithm [106]. Unlike miRbase that solely stores the genomic features, miRTarBase also collects experimentally validated miRNA-target information [107, 108]. The DIANA Lab (http://diana.imis.athena-innovation.gr/DianaTools/index.php) focuses on investigating the genomic sequences and functions of ncRNAs, which motivates the development of multiple algorithms, databases and software. DIANA-microT-CDS tool, evolved from microT algorithm, implemented machine learning methods including SVM, neural networks, random forests, and generalized linear models to calculate the score of MREs located in both the 3’-UTR and CDS regions of the target gene [109]. DIANA-TarBase provides miRNA-target across multiple species, cell types and tissues regarding its compilation of experimental data supported by ~600 cell types/tissues [110]. miRWalk, an online platform, integrated with TarPmiR algorithm to primarily predict from 3’-UTR, 5’-UTR, and CDS, followed by validation using eight other tools including TargetScan and miRanda *etc.* [111, 112]. doRiNA database is built based on PicTar miRNA-target prediction software, which serves *via* web server to predict the candidate sites by probability score on the seed matching [113].

### Tools for circRNA-miRNA-mRNA prediction

For fast and easy-use, some programs have been developed with integration of multiple tools and databases to construct circRNA-miRNA-mRNA axis in one program. starBase collects and retrieves sequence data from miRbase, and predicts the ceRNA network using TargetScan, miRanda, PITA, RNA22, and Pictar [114]. CircMiMi and CRAFT enable users to find miRNA-mRNA network with the corresponding input circRNA coordinates by using miRanda, PITA, miRDB, miRTarBase, circAtlas *etc.* for search [115, 116]. While CircMiMi provides extensive ceRNA networks interactions across 16 mammalian and non-mammalian animals and two plant species, intensive computational skills are necessitated due to its nature of machine learning-based approach.

### Examples of predicted ceRNA networks and their experimental validation

While the number of tools and databases have been developed for ceRNA networks prediction, the experimental validation is also necessary to enhance the reliability and accuracy of the predicted network. Zhang et. al conducted the prediction of circRPPH1/hsa-miR-326 using the CircInteractome database, which was validated using dual luciferase reporter activity, revealed the potential sponge effect of circRPPH1 on miR-326 [117]. They also applied several miRNA-specific databases including miRTarBase, miRBD, and TargetScan to predict that integrin subunit alpha 5 (ITGA5) might be the target gene of miR-326, which was also validated using western blot and qRTG-PCR. This study summarized that circRPPH1 negatively regulates miR-326 and up-regulates ITGA5 expression through ceRNA interactive axis. Among Table 2, CPAT and TargetScan were involved in the study that predicts the lncRNA Mt1JP/miR-92a-3p/FBXW7 axis, which was also validated using laboratory techniques to identify the competitive binding of the lncRNAs and mRNAs during post-transcriptional regulation [118]. An intensive studies have demonstrated that the utility of computational approaches benefit the scientific research from high-speed scanning by providing a considerably reliable predictive outcomes, subsequently reinforming the applicability of these tools and databases.

## Challenges and future directions

In the recent decade, the number of predicted ncRNAs generated is largely a consequence of high-throughput RNA-seq technology and bioinformatics tools. Although advanced software has provided a drastic increase in identification and annotations for transcripts, challenges arise due to the biological complexities, technical limitations, and computational constraints.

Across circRNAs detection tools, detection of back-splice junctions (BSJs) is the most important feature for predicting circRNAs presence. Therefore, alignment rate which is regulated by misalignment or ambiguous alignment results in the level of false positives in BSJ detection [126]. An evaluation study revealed that artificial junction formation could be caused by template switching during the reverse transcription reaction, that eventually produces false BSJs as a result [127]. Although circRNAs are resistant to RNase R degradation due to its circular structure, misidentification of circRNAs can occur as some linear RNAs with G-quadruplex structures are also RNase R resistant [128]. Additionally, long circRNAs could be more sensitive to RNase R degradation if the treatment was contaminated by endonucleases [129]. Different algorithms commit to their computational bias, either for higher accuracy or sensitivity through manipulating the gene annotations or canonical U2 splice signals [130]. As some parameters such as filtering criteria and alignment setting are usually set up by project researchers, this modification motivates the circRNAs detection algorithm to become sensitive to the performance. Since it is widely known that the preparation of RNA-seq data requires multiple complex conditions and procedures, the lack of a universal criteria to restrict the quality of RNA-seq datasets that are used as references for circRNAs annotations could lead to a misinterpretation of the predicted transcripts [131]. Therefore, high RNA integrity (RIN), efficient depletion of rRNA and sufficient sequencing depth of coverage are examples of critical measurements for RNA-seq data preparation and collection [26, 132]. By regulating the quality criteria, investigation projects that are based on RNA-seq datasets would embrace more accurate reference to RNA molecules, eventually improving the predictions of circRNA transcripts and discovery of interactions between RNA molecules using RNA-seq datasets.

Although the combination of deep learning models and RNA-seq elevates efficiency and performances for lncRNAs detection, limitations still exist with transcript identification [87]. The biogenesis and genomic sequences of lncRNAs and mRNAs share many similarities such as transcript size and transcription of genomic loci through RNA polymerase II [133]. Some crucial features are selected to differentiate lncRNAs from mRNAs, including the fewer but longer exons, shorter length of ORFs and comparatively lower expression levels [134]. However, each tool has been trained with bias to perform more satisfactorily on one or more, but not all features. Regarding the distinct secondary structure of lncRNAs, this local spatial conformation has not been accounted in a majority of detection algorithms (Table 2). The evaluative study compared the performance of algorithms through training machine learning models with annotation datasets and context-specific annotation datasets [87]. Annotation datasets denote the collective information on gene structure, sequence and functions, and context-specific annotation datasets denote the expression of transcripts in specific cell types. Although the good performance from these machine learning tools was achieved with annotation datasets, they reported that tools dropped to poor performance with context-specific datasets.

Tools and databases for miRNAs identification and miRNA-target predictions are limited to sequencing errors and technical restrictions. Challenges have been shown in capturing novel miRNAs through RNA-seq, and the low-quality of RNA-seq sequences could seriously affect the miRNAs quantification [107]. It was noted that the degraded RNA fragments during sequencing could be miRNAs [135]. While the sequence length of miRNAs is very small, there are some miRNAs expressed at low levels in temporally-, tissue- and disease-specific manners, these nuances make miRNAs more difficult to identify [136]. To date, miRNA-targets are predicted based on complementary binding of MREs at seed sequence position 2–7, interaction outside this region becomes complicated in prediction [100, 105]. Evaluation of the secondary structure and target site accessibility is also vital which can possibly generate non-functional interaction or miss the potential miRNA-mRNA interaction [109].

The overall expectation with high-throughput and advanced technology is to combine multiple technologies and approaches to improve performance and minimize false-positive results [87, 136, 137]. In previous studies, laboratory techniques are adopted to validate the predicted interaction between ceRNAs and miRNAs [42, 52, 53, 63]. Qualitative reverse transcription polymerase chain reaction is widely used to evaluate the expression levels of the predicted RNA species. Fluorescence *in situ* hybridization is used for identification of colocalization of the predicted RNA molecules. Luciferase reporter assay is commonly performed for detection of the interaction between the ceRNAs and miRNAs. Implementation of more robust filters can be necessary to remove artifacts initiated by template switching in circRNAs detection technique. A standardized criteria can maintain circRNA annotations for consistency across the datasets. In the lncRNA detection pathway, incorporation of a wide range of features including genomic structure, species-specific differences, and consistent annotations would improve the performance of algorithms. Utilisation of high-throughput technology such as CLIP-seq, PAR-CLIP, and AGO-RIP can further validate the predicted miRNA-target interaction [138]. Advanced scRNA-seq (single cell RNA-seq) could provide greater understanding on the correlation between ceRNAs and miRNAs through measuring the transcriptional expression in cells [139–141]. Spatial multi-omics approaches are also option to explore the perspective of regulatory network while predicting circRNA/lncRNA-miRNA-mRNA axis [142, 143]. Scrutinisation of future advancement and the relevance of ceRNAs and disease progression would benefit from using scRNA-seq to explore distinct cell-type-specific ceRNAs networks across diverse species, along with diving into the insights into dynamic the interactions ceRNAs in contexts of tissue-specific expression using spatial multi-omics.

## Conclusion

This review covered the complex biogenesis within the competitive endogenous RNAs, emphasising the regulatory roles of circular RNAs, long non-coding RNAs, and microRNAs. We also highlighted the function of the circRNA/lncRNA-miRNA-mRNA network in various disease and biological pathways. Several bioinformatics tools which were introduced in this review have been accelerating the exploration of such ceRNAs network. Although the biological concepts behind ceRNAs across all species and tissues remain obscure, further improvement of technologies could unveil the complexity of ceRNAs in biological systems.

**Key Points**

- Non-coding RNAs (ncRNAs) are classified on length and function, primarily into small ncRNAs, long RNAs and circular RNAs.
- ncRNAs and mRNAs that compete for binding to micro RNAs have been termed as competitive endogenous RNAs (ceRNAs).
- ceRNA-miRNA network significantly impact on miRNA-mediated gene expression such as translation repression and mRNA decay.
- ceRNA-miRNA network play a crucial role in disease development by functioning on the biological and physiological regulations.
- Computational tools and databases can be used to identify and predict the ceRNA-miRNA interactions.
